# 5-Azacitidine Exerts Prolonged Pro-Apoptotic Effects and Overcomes Cisplatin-Resistance in Non-Seminomatous Germ Cell Tumor Cells

**DOI:** 10.3390/ijms20010021

**Published:** 2018-12-21

**Authors:** Christoph Oing, Izudin Verem, Wael Y. Mansour, Carsten Bokemeyer, Sergey Dyshlovoy, Friedemann Honecker

**Affiliations:** 1 Department of Oncology, Hematology and Bone Marrow Transplantation with Division of Pneumology, University Medical Center Eppendorf, 20246 Hamburg, Germany; c.oing@uke.de (C.O.); izudinverem@googlemail.com (I.V.); c.bokemeyer@uke.uni-hamburg.de (C.B.); s.dyshlovoy@uke.de (S.D.); 2 Laboratory of Radiobiology and Experimental Radiation Oncology, University Medical Center Eppendorf, 20246 Hamburg, Germany; w.mansour@uke.de; 3 Tumor Biology Department, National Cancer Institute, Cairo University, Cairo 11796, Egypt; 4 Laboratory of Marine Natural Products Chemistry, G.B. Elyakov Pacific Institute of Bioorganic Chemistry, Far-East Branch, Russian Academy of Sciences, 690022 Vladivostok, Russian Federation; 5 School of Natural Sciences, Far Eastern Federal University, 690000 Vladivostok, Russian Federation; 6 Tumor and Breast Center ZeTuP St. Gallen, CH-9006 St. Gallen, Switzerland

**Keywords:** germ cell tumor, 5-azacitidine, DNA demethylation, DNA methyl transferase (DNMT) inhibition, cisplatin resistance

## Abstract

Despite high cure rates, about 20% of patients with advanced germ cell tumors (GCTs) fail cisplatin-based chemotherapy. High levels of DNA methylation have been identified in GCTs and linked to cisplatin resistance. Here, we examined the effects of DNA hypomethylating 5-azacitidine (5-aza) on two embryonal carcinoma cell lines (NCCIT, 2102Ep) and their cisplatin-resistant isogenic derivatives. Effects on cell viability and cisplatin sensitivity were assessed by the trypan blue exclusion method. Western blotting was used to examine induction of apoptosis 5-aza and results were validated by flow cytometry. Single agent treatment with 5-aza strongly impacted viability and induced apoptosis at low nanomolar concentrations, both in cisplatin-sensitive and -resistant cell lines. 5-aza exerted an immediate apoptotic response, followed by a prolonged inhibitory effect on cell viability and cell-cycle progression. Sequential treatment with 5-aza and cisplatin reduced cellular survival of the cisplatin-resistant sublines already at nanomolar concentrations, suggesting a partial restoration of cisplatin sensitivity by the compound. 5-aza demonstrated anti-tumor activity as a single agent at low nanomolar concentrations in GCT cells, irrespective of cisplatin-sensitivity. 5-aza may also have the potential at least to partially restore cisplatin-sensitivity in non-seminoma cells, supporting the hypothesis that combining DNA demethylating agents with cisplatin-based chemotherapy may be a valid therapeutic approach in patients with refractory GCTs.

## 1. Introduction

Germ cell tumors (GCTs) are the most common solid malignancy among men up to the age of 45. Histologically, GCTs are classified into undifferentiated, primordial germ cell-like seminomas and more differentiated non-seminomas. Based on the extraordinary sensitivity towards cisplatin-based chemotherapy, GCTs are recognized as a model of a curable disease, even in advanced, metastatic stages [1]. However, about 30% will fail first-line platinum-based combination chemotherapy, of which only 50% will achieve long-term survival after salvage treatment [2]. Thus, about 15–20% of advanced-stage GCT patients must be considered platinum-refractory, putting them at risk of succumbing to the disease. Despite many efforts to improve outcomes in this situation, the therapy efficacy remains rather poor [3]. The multiple mechanisms underlying cisplatin resistance are still incompletely understood [4]. However, there is a growing body of evidence that epigenetic alterations, i.e., gene promoter hypermethylation, are related to cisplatin-resistance [5]. In general, there is an unmet need for predictive biomarkers and new treatment options to improve treatment success i.e., by overcoming treatment resistance.

Epigenetic modifications play a significant role in carcinogenesis and in response of cancer cells to chemotherapy through regulating the expression of genes involved in cell replication, tumor suppression, DNA repair and apoptosis [6]. Epigenetic changes comprise (i) DNA methylation, (ii) histone post-translational modifications, (iii) nucleosome remodeling, and (iv) microRNAs as post-transcriptional gene regulators [7,8]. Methylation of cytosine residues is a major regulatory mechanism. This process is catalyzed by three different enzymes called DNA methyl transferase (DNMT), which maintains a cells methylation pattern during mitosis, as well as DNMT3A and DNMT3B, which are responsible for de novo 5mC formation [9]. The DNA methylation pattern of a cell strongly interferes with its gene expression profile and biological behavior. Like primordial germ cells and cells of the tumor precursor germ cell neoplasia in situ (GCNIS), seminomas have been shown to contain a completely unmethylated genetic makeup. While the least differentiated non-seminomatous subtype embryonal carcinoma shows an intermediate DNA methylation pattern, other subtypes with a higher degree of differentiation are generally hypermethylated [10,11]. Furthermore, promoter hypermethylation has previously been reported to contribute to cisplatin resistance in GCTs [5,11,12]. As a reversible epigenetic modification, DNA hypermethylation can be targeted therapeutically.

Chemical DNA demethylation can restore the expression of silenced genes, which can be achieved, for example, by the use of the first generation DNMT inhibitor (DNMTI) 5-azacitidine (5-aza), which is approved by the Food and Drug Administration (FDA) for the treatment of myelodysplastic syndromes. The cytotoxic activity of 5-aza as a ribonucleoside, which gets incorporated into both DNA and RNA, is based on broad effects on both DNA methylation patterns and the cell’s transcriptome and gene expression profile [13,14]. In a seminoma cell line, 5-aza lead to a decrease in cisplatin resistance [11]; possible molecular targets involved in this phenomenon where changes in gene expression, i.e., demethylation of *KLF11*, a putative tumour suppressor gene, and hypermethylation of *CFLAR*, a gene previously described in treatment resistance in GCTs.

Here, we examined the effect of 5-aza, which has not been examined in non-seminomatous GCT cells so far, on two distinct embryonal carcinoma (EC) cell lines, e.g., NCCIT bearing a mutant *TP53*, and 2102Ep, which has wild type *TP53,* in an in vitro model system of acquired cisplatin-resistance using isogenic, resistant sublines NCCIT-R and 2102Ep-R.

## 2. Results

### 2.1. Embryonal Carcinoma (EC) Cells are Highly Sensitive to 5-Aza at Nanomolar Doses Irrespective of Cisplatin-Sensitivity

**Figure 1:**
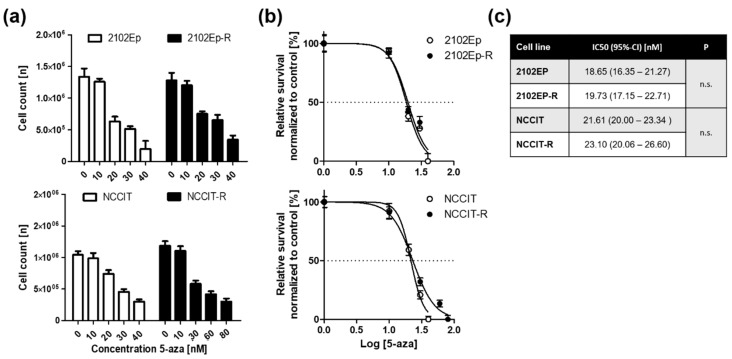
Embryonal carcinoma (EC) cell lines are very sensitive to nanomolar doses of 5-aza. 5-aza was added at the indicated concentrations over a 72 h-period and replenished each day. Viable cells were assessed by trypan blue exclusion method. Means of three identical experiments are displayed. Each experiment was conducted at least three times with similar results. (**a**) Absolute cell counts. (**b**) Normalized inhibitory response curve to 5-aza. (**c**) IC_50_ values calculated by non-linear regression analysis.

At first, the sensitivity of the cell lines 2102Ep and NCCIT and their cisplatin-resistant, isogenic sublines 2102Ep-R and NCCIT-R towards the DNA demethylating agent 5-aza was measured by Trypan blue assay and the respective IC50 values were determined by non-linear regression for each cell line (Figure 1a,b). Basically, our results revealed that cell viability in all 4 tested cell lines—irrespective of their cisplatin-sensitivity—was strongly reduced after 72 h of repeated 5-aza exposure with IC_50_ values ranging from 18 to 23 nM (Figure 1c).

### 2.2. Exposure to Nanomolar Concentrations of 5-Aza Induces a Strong and Prolonged Apoptotic Response in EC Cells 

**Figure 2:**
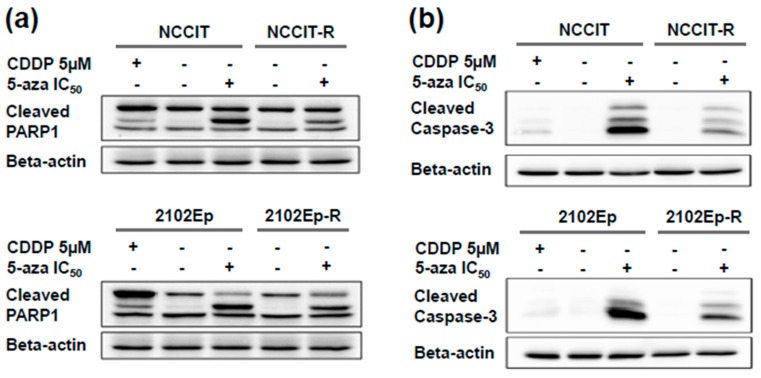
Nanomolar 5-aza treatment causes apoptosis induction in all four tested cell lines. Both (**a**) PARP1 cleavage, and (**b**) caspase-3 cleavage occur after 72 h of treatment with the respective IC_50_ of 5-aza. Graphically, the amount of cleaved protein appears slightly decreased in the isogenic cisplatin-resistant sublines NCCIT-R and 2102Ep-R when compared to their sensitive counterparts. 5-aza is a strong inductor of apoptosis. Cells treated with 5µM cisplatin (CDDP), a supralethal dose, served as positive controls for the induction of apoptosis.

The effect of 5-aza treatment on apoptosis in EC cells was assessed. To that end, cells were treated with the corresponding IC_50_ doses of 5-aza for 72 h and apoptosis was analyzed by monitoring the cleavage of Caspase-3 and Poly-(ADP-ribose) polymerase 1 (PARP1). The cisplatin sensitive EC cells treated with their respective IC_50_s of cisplatin for 72 h served as controls of apoptosis induction. In all four cell lines we detected a strong apoptotic response upon 72 h of treatment with the respective IC_50_ doses of 5-aza as single agent as evidenced by increased caspase-3 and PARP1 cleavage (Figure 2a,b). Interestingly, bands of both cleaved proteins showed stronger intensity upon 5-aza treatment as compared to single agent cisplatin treatment, and the levels of cleaved proteins were higher in the cisplatin-sensitive parental cell lines (Figure 2a,b). 

**Figure 3:**
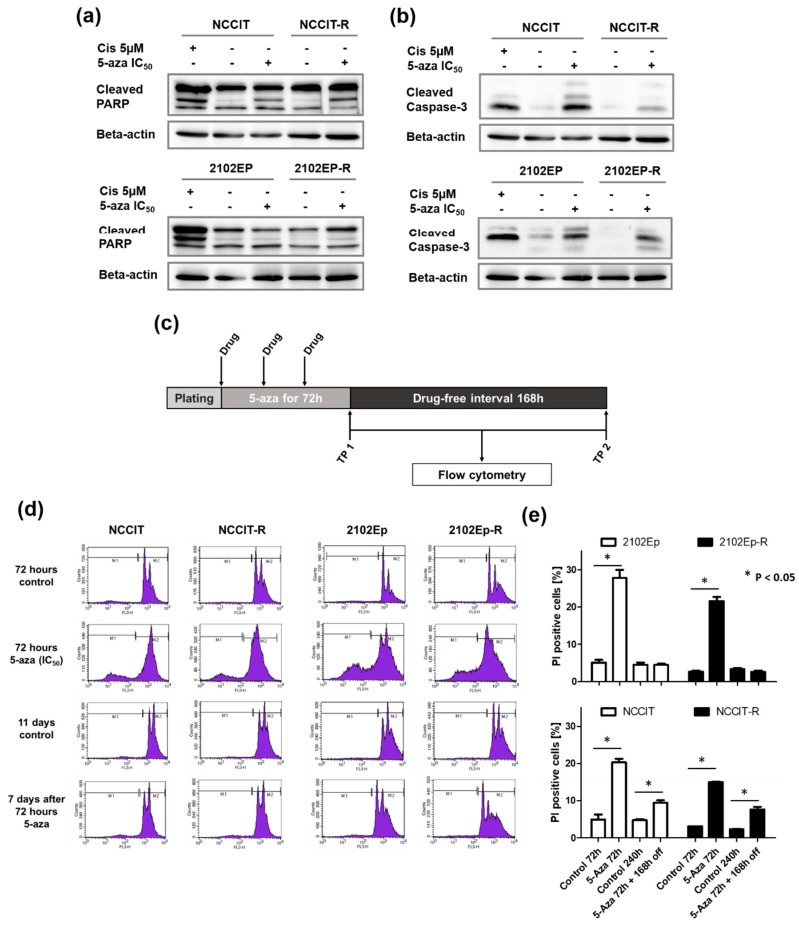
5-aza treatment exerts sustained apoptotic impact on EC cell lines. (**a**) PARP1 cleavage remains high in NCCIT and NCCIT-R cells, while 2102EP and 2102EP-R cells seem to lose PARP1 degradation over time. (**b**) Caspase-3 cleavage continues in all 4 cell lines, but to a lesser extent in both cisplatin-resistant isogenic sublines. Interestingly, after a 168 h-break after 5-aza exposure, 5µM cisplatin seemed to induce delayed apoptosis to the highest degree. (**c**) Schematic experimental design of cell cycle analyses (TP, time point). (**d**) Flow cytometric analysis of PI-positive apoptotic cells among all tested cell lines. (**e**) Evaluation of the experiments conducted in D. Even a week after the end of 5-aza treatment, *TP53*-mutant NCCIT and NCCIT-R cells underwent apoptosis more frequently when compared to untreated control cells. Shown are mean ± standard deviation (SD) of experiments, which were repeated three times with similar results.

Subsequently, a prolonged cultivation of cells after drug exposure to 5-aza was applied to achieve a maximum effect of the drugs’ acitivity since demethylation is expected to require several cell doublings for 5-aza incorporation into the DNA strands. Following a 168 h drug-free period after 5-aza treatment, pro-apoptotic activity was still substantial in both the pluripotent, *TP53*-mutated cell lines NCCIT/-R and the nullipotent, p53-wild type cell lines 2102Ep/-R (Figure 3a,b). Again, the paternal cisplatin-sensitive cells exposed to 5 µM cisplatin over 48 h served as positive control and similarly showed a sustained impact on apoptosis induction, which as expected was stronger after the 7-day break (Figure 3a,b). This suggests a prolonged pro-apoptotic effect exerted by 5-aza, which seems independent of p53-signaling, however, this speculation awaits further confirmation. 

The effect of 5-aza on apoptosis was further recapitulated by employing flow cytometry using PI staining to detect the subG1 cell population immediately and 168 h after short-term 72 h treatment with 5-aza at the respective IC_50_ doses (Figure 3c). Again, there was a strong apoptotic response immediately after 72 h of 5-aza exposure among all four tested cell lines. A significant sustained pro-apoptotic effect was only detected by FACS for the p53-mutant NCCIT/-R cells (Figure 3d,e). Taken together, this indicates that irrespective of cisplatin-resistance and *TP53*-mutational status, all cell lines responded with a sustained apoptosis induction. 

### 2.3. Nanomolar Concentration of 5-Aza Overcomes Cisplatin-Resistance in EC cells

**Figure 4:**
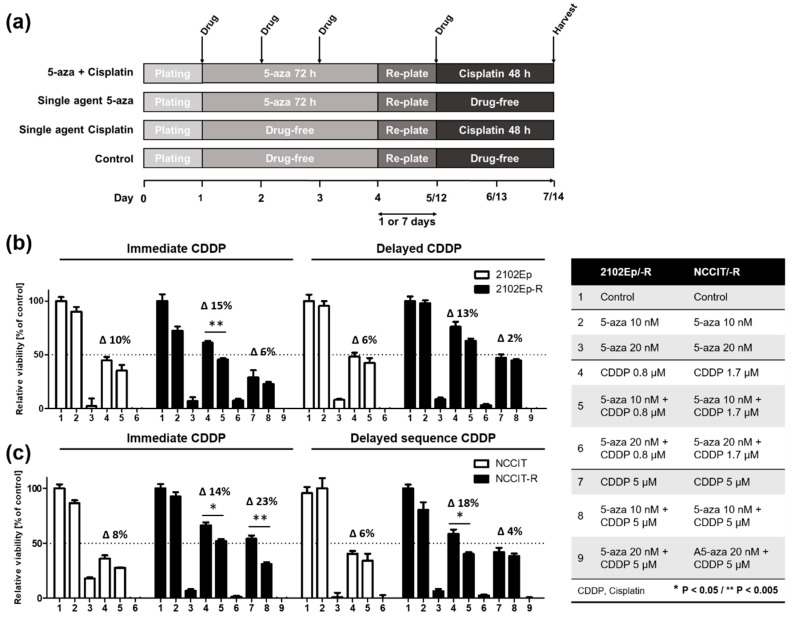
Cell viability assay (**a**) showing enhanced toxicity of 72 h exposure to 20nM 5-aza (~IC_50_) plus 72 h off treatment among all cell lines, 2102Ep/-R and NCCIT/-R (**b**,**c**). A significant sensitization towards a regular dose of cisplatin by adding a low dose of 5-aza (10 nM) was achieved in both resistant sublines by either immediate or delayed sequential treatment decreasing viability by 13–18%. This effect was less clear and not significant in the sensitive cell lines. Shown are mean and SD of a single triplicate experiment. Each experiment was conducted at least three times with similar results. CDDP, Cisplatin; ∆, numeric difference between relative viabilities (%).

The levels of cisplatin resistance in the individual cell lines have been determined previously [15]. Here, we sought to define whether 5-aza would sensitize cisplatin-resistant cells to cisplatin. Therefore, the cells were first exposed to 5-aza for 72 h at either a low concentration of 10 nM or the respective IC_50_, followed by a 48 h of cisplatin treatment at either the respective IC_50_ of the sensitive cell lines NCCIT (1.7 µM) or 2102Ep (0.8 µM) or a supra-lethal dose of 5 µM representing the IC_50_ of both resistant sublines NCCIT-R and 2102Ep-R. With respect to a possible impact of the timing of cisplatin exposure after 5-aza treatment, two different schedules were tested. Cisplatin was applied either 24 h after re-plating (immediate sequence), or after a 168 h drug-free period following 5-aza exposure (delayed sequence) to assess possible prolonged effects of 5-aza on cisplatin sensitivity (Figure 4a). Viability was assessed using the Trypan blue assay. 

As expected, single agent cisplatin exposure at the IC_50_ doses of the cisplatin-sensitive paternal cell lines (0.8 µM and 1.7 µM for 2102Ep and NCCIT, respectively) was significantly less effective in the isogenic cisplatin-resistant sublines with a mean relative viability of 38% vs. 63% (*p* ≤ 0.0001) for NCCIT/-R and 46% vs. 69% (*p* = 0.0007) for 2102Ep/-R. Moreover, cisplatin demonstrated a dose-dependent toxicity in the resistant sublines after 48 h of treatment with either the aforementioned doses or a supra-lethal dose, showing a 14% reduction of the mean viability for NCCIT-R (*p* = 0.0091) and a 30% for 2102Ep-R (*p* = 0.001) (Figure 4b,c). Together, this data validates the resistant phenotype in the resistant subclones.

Treatment with 10 nM 5-aza alone did not significantly impact cell viability when compared to untreated controls, confirming the results in Figure 1a,b. Notably, treatment with 20 nM, which almost equals the respective IC_50_ doses of 5-aza for all cell lines, decreased cell viability to an unexpectedly high extent compared to the experiments shown in Figure 1. This may be explained by a sustained impact on cell viability of 20 nM 5-aza during the prolonged drug-free culturing period of an additional 72 h, which were needed for the combined treatment with cisplatin as a comparator.

Consistently, combining cisplatin with 20 nM 5-aza had the most profound effect in all cell lines tested. Therefore, we sought to use a lower dose of 5-aza (10 nM), which did not show significant impact on cell growth (as shown in Figure 2a,b), for further combination experiments. Interestingly, combined treatment with 5-aza at a low dose of 10 nM for 72 h and a subsequent 48 h cisplatin exposure reduced cell viability more effectively than either single agent treatment in all tested cell lines. A stronger and statistically significant effect of the combined sequential treatment compared to cisplatin alone was detected among the cisplatin-resistant sublines 2102Ep-R (Figure 4b, black bars) and NCCIT-R (Figure 4c, black bars). Viability was significantly reduced upon adding cisplatin at the IC_50_ of the corresponding sensitive cell lines, ranging from 14% (*p* = 0.012) to 18% (*p* = 0.014) in NCCIT-R cells, and from 15% (*p* = 0.019) to a borderline significant 13% (*p* = 0.058) in 2102Ep-R cells for immediate and delayed sequencing, respectively. Importantly, combined treatment decreased cell viability in the resistant sublines almost to levels achieved by cisplatin alone in the sensitive cell lines (Figure 4b,c, white bars; column 4) (*p* = 0.21). Except for the cisplatin-resistant, *TP53*-mutant subclone NCCIT-R (23% reduced viability, *p* = 0.0025), combining 10nM of 5-aza with a supra-physiological dose of cisplatin (5µM) did not add a relevant cytotoxic effect compared to 5µM of cisplatin alone (Figure 4b,c; columns 7,8). With respect to the timing of cisplatin exposure after 5-aza treatment, there was no clear signal favoring either the immediate or delayed approach pointing towards a sustained sensitizing effect of 5-aza.

## 3. Discussion

Cisplatin resistance is a rare but clinically challenging situation in GCT patient care. Cisplatin is the most effective chemotherapeutic agent in the treatment of advanced and relapsed GCTs. It is commonly applied as part of combination chemotherapy regimens with a standard dosage of 20 mg/m^2^ daily on days 1 to 5, every 3 weeks. Maximum platinum plasma concentrations have been reported to be as high as 0.6 to 14 µM, with a median of 3.3 µM [16]. Cisplatin-resistant disease is most likely of multifactorial origin [4]. Cisplatin-refractory patients failing several lines of systemic combination chemotherapy, including high-dose chemotherapy, have a very poor prognosis. Therefore, there is an urgent need to better understand and eventually overcome resistance mechanisms [3]. 

Both cell lines used in this project originate from embryonal carcinomas, a common chemo-sensitive subtype of non-seminomatous GCTs. The cell line NCCIT is derived from a primary mediastinal germ cell tumor with p53-deficiency, as it is frequently found in these rare tumors. *TP53* gene mutations and alterations in related pathways (i.e., by MDM2 aberrations) are thought to be one mechanism leading to cisplatin resistance in GCTs, mediated by an altered apoptotic response to treatment [17,18]. Importantly, the cisplatin concentrations used in our in vitro cell line model system reflect physiologically (in vivo) achievable plasma levels with respect to the IC_50_ values for the sensitive lines NCCIT and 2102Ep. The IC_50_ of the resistant sublines was much higher at supra-physiological 5 µM as reported before, which thus appropriately reflects the clinical burden of cisplatin-resistant disease [15]. Thus, studying these cell lines may facilitate translation of the in vitro results even into clinical applications, in the future.

Epigenetics refers to gene expression alterations without changes in the DNA sequence. The most intensively studied epigenetic mechanisms comprise DNA methylation, regulation by microRNAs, and covalent, post-translational histone tail modifications, i.e., methylation and acetylation [7]. The different GCT subtypes display a different extent of promoter hypermethylation. Seminomas, which are clinically the most sensitive subtype, generally show hypomethylation, whereas the more frequently treatment-resistant non-seminomas show strong hypermethylation, most obviously demonstrated by high levels of hypermethylation found in differentiated teratomas. The other non-seminoma subtypes, embryonal carcinoma, choriocarcinoma and yolk sac tumors show intermediate, subtype specific methylation patterns [11,19,20]. Promoter hypermethylation has been described to contribute to chemotherapy resistance in testicular GCTs, particularly hypermethylation of the *RASSF1A*, *HIC1* and *MGMT* promoters [5,11,12]. 

Aberrant DNA hypermethylation is a reversible epigenetic alteration, which can be therapeutically targeted with the aim of achieving global DNA hypomethylation and re-expression of tumor-suppressor genes [11,21]. 

Overall, our results, where single agent 5-aza was equally effective in both p53-deficient (NCCIT) and proficient (2102Ep) isogenic in vitro models of cisplatin-resistant GCTs, are in line with those reported for the treatment of the embryonal carcinoma cell line NT2/D1 and its resistant subline NT2/D1-R1 with either decitabine or guadecitabine. Moreover, sensitivity towards therapeutic DNA demethylation with either decitabine or guadecitabine was functionally dependent on a high expression of DNMT3B [12,21]. This may also be true for 5-aza; however, it remains to be elucidated to date. Importantly, all three agents, 5-aza, decitabine and guadecitabine, actively impact GCT cell line survival at low nanomolar concentrations irrespective of the cisplatin-resistance level of the different cell lines tested (NCCIT, 2102Ep and/or NT2/D1). Functionally, low-dose decitabine and guadecitabine have been reported to induce a p53 transcriptional signature, which is distinct from that related to cisplatin exposure, and to repress the transcription of pluripotency genes, i.e., *NANOG* or *SOX2*, and Myc target genes in NT2/D1 cells [21,22,23]. On the contrary, Wermann et al. reported an increased expression of the pluripotency markers *NANOG* and *OCT3/4*, or hypermethylation of distinct putative drivers of cisplatin-resistance, i.e., *CFLAR*, together with decreased cisplatin resistance upon 5-aza treatment in the seminoma cell line TCam-2 [11]. Taken together, DNMTI seems to have different, sometimes even contradictory modes of action, but to finally be active in various GCT cell line models. Importantly, in our study the detrimental effect of 5-aza was significant in the cisplatin-resistant isogenic subclones and independent of *TP53*-mutation status, which is why our results add another important aspect to the complexity by showing a sustained strong apoptotic response and a (at least partial) restoration of cisplatin sensitivity irrespective of the cell lines *TP53* mutational status (*TP53* mutated NCCIT, *TP53* wild-type 2102Ep). This observation supports the hypothesis that the effects of DNMT inhibition may be multi-facetted, which could mean that the majority of cisplatin-resistant GCTs may be amenable to 5-aza (and/or other DNMTI) treatment irrespective of key clinical aspects related to cisplatin-resistance, particularly p53 pathway aberrations [18].

In general, GCTs consistently displayed an extraordinary sensitivity towards DNA demethylating drugs at low nanomolar concentrations, which is why DNA demethylation, either alone or in combination with other cytotoxic substances, holds promise as a new treatment strategy in refractory GCTs.

Being the first and clinically the most often used epigenetic drug so far, 5-aza is currently recommended as first-line treatment of high-risk myelodysplastic syndromes and acute myeloid leukemia in patients unsuitable for more aggressive chemotherapy. Repeated cycles of 5-aza have proven to be highly effective and well tolerated in such patients [24,25,26]. An early clinical trial evaluating high-dose 5-aza in refractory GCT patients failed to achieve any favorable responses, but reported remarkable toxicity [27]. DNMTIs mimic cytosine residues and get incorporated into the DNA strand during DNA synthesis, which leads to trapping of DNMTs on the newly synthesized DNA, blocking their function and promoting degradation of the DNA-protein adducts. Eventually, this leads to global DNA hypomethylation, which enables the re-expression of genes, among others tumor suppressor genes, causing an overall anti-neoplastic effect [28]. This mode of action may increase over several cell cycles, which is why prolonged exposure to lower DNMTI doses is crucial for the anti-neoplastic effect of DNMTIs, such as 5-aza. High doses of 5-aza, instead, exert an unspecific cytotoxic effect, which has been shown to be ineffective in solid tumors [26,27].

The standard dosage to treat hematological malignancies is a subcutaneous injection of 75 mg/m^2^ daily, days 1 to 7, every 4 weeks, with a reported peak plasma concentration 750 ± 403 ng/mL occurring 0.5 h after infection with a half-life of 41 ± 8 min. A slightly different but also intense dosage was applied in the phase II trial by Roth et al. (150 mg/m^2^ per day, days 1 to 5, every 3 weeks) evaluating 5-aza as a single agent in 17 refractory GCC patients, where no responses were seen [26]. With respect to the molecular weight of 5-aza (244.2 g/mol), a peak plasma concentration of 750 ng/mL equals 3.071 µM. Although cell culture conditions are hard to translate into in vivo observations, the doses of 10 to 20 nM 5-aza applied in our in vitro experiments are thus significantly lower (0.3–0.6% of the reported peak plasma levels) than those reported to induce relevant clinical responses in hematologic malignancies and also far below the cytotoxic concentrations used in the only GCT trial. 

Hypothetically, much lower doses may thus be effective in vivo to (at least partially) overcome cisplatin-resistance in GCTs and to reduce toxic side effects at the same time. Interestingly, the IC_50_ determined in EC cell lines in this study, as well as those reported for other DNMTIs in the literature, are much lower and more in the range found in hematological neoplastic cells than the doses causing anti-neoplastic effects in solid cancer models when using other entities, such as pancreatic, hepatocellular, or breast cancer cells [29,30,31]. Lower doses seem to exert a different molecular mode of action and have the advantage of a substantially improved safety profile. Moreover, we provide in vitro evidence that the cisplatin-sensitizing effect of 5-aza is sustained at least over a one week-period. This underlines that both dosage and application mode should be thoroughly considered to enhance the clinical efficacy of DNA demethylating agents.

Presumably due to the multifactorial origin of platinum-resistance, targeted approaches using single agents failed to achieve clinically meaningful responses in refractory GCTs so far. Combined therapeutic approaches may thus be more promising [3]. Interestingly, the use of continuously applied low doses of 5-aza-2’-deoxycitidine, decitabine (DAC) followed by carboplatin was reported to transiently re-sensitize heavily pretreated patients with platinum-insensitive ovarian cancer to carboplatin, as documented by a 35% response rate and a significant median progression-free survival interval of 10.2 months in a phase II study [32]. A phase I trial reported similarly favorable responses for the combination of guadecitabine in platinum-resistant ovarian cancer [33]. Additionally, guadecitabine has been shown to re-sensitize ovarian cancer cell lines towards cisplatin both in vitro and in a xenograft model [34]. Although mechanisms of platinum-resistance are not fully understood and may differ between ovarian and testicular cancer, these results are encouraging and worthy of being further explored in cisplatin-resistant GCTs as well. Currently, there is one ongoing phase I trial from the Indiana University Hospital, US, investigating the combination of guadecitabine and cisplatin in patients with refractory GCTs (see clinicaltrials.gov, Identifier NCT02429466), and others are likely to follow.

## 4. Materials and Methods 

### 4.1. Cell Lines and Culturing Conditions

The extragonadal embryonal carcinoma cell line NCCIT was purchased from the American Type Culture Collection (ATCC; Manassas, VA, USA). The gonadal embryonal carcinoma cell line 2102Ep was a generous gift from P.W. Andrews (Centre for Stem Cell Biology, The University of Sheffield, Sheffield, UK). Cells were routinely checked for contamination with mycoplasma and inspected microscopically for stable phenotype. All the cell lines used in the current research have been tested and authenticated.

Both cell lines were maintained in Dulbecco’s modified Eagle medium (DMEM)/F12, 1:1 nutrient mixture medium (Life Technologies; Carlsbad, CA, USA) with 10% fetal bovine serum supplement (Gibco, Invitrogen, UK) plus L-Glutamine (2 mM) (Sigma-Aldrich, St. Louis, MO, USA), and 50 IU/mL penicillin and 50 µg/mL Streptomycin (Gibco, Grand Island, NY, USA). For both cell lines, isogenic, cisplatin-resistant subclones were developed by repeated exposure to sublethal, increasing cisplatin-doses as described before [15,35]. Cells were kept in an incubator at 37 °C in 5% CO_2_. Cells were passaged when reaching 80–90% confluency, and only used for assays in passages 3–7. 

### 4.2. Cytotoxic Treatment

5-azacitidine (5-aza) was purchased from Selleckchem (Cat.-No. S1782; Absource Diagnostics GmbH, Munich, Germany). For the treatment, 5-aza was freshly dissolved in phosphate-buffered saline (PBS) and added daily to the culturing medium at the indicated concentrations for 72 h with drug-containing media replenishment each day. Cisplatin solution was a generous gift from Medac Pharma (Wedel, Germany). Cisplatin treatment was performed at the indicated concentrations and time points for 48 h. 

### 4.3. Cell Viability Assay

For assessment of cell viability, 1.5 × 10^5^ cells/well were seeded in 6-well format in complete growth medium (2 mL/well). Following the plating, the cells were incubated overnight to allow proper adhesion before drug treatment. For determination of the IC_50_ for 5-aza, cells were treated with different doses of the drug in triplicate for 72 h. Cell viability was assessed by Trypan blue staining method combined with the automatic cell counting using Beckman Coulter Vi-CELL (Beckman Coulter, Krefeld, Germany). For combined treatment, two approaches were applied: (i) cells were treated with 5-aza at either a fixed-dose of 10 nM, or the respective IC_50_ over 72 h. Cells were then washed with PBS, trypsinized, pelleted using centrifugation at 300× *g*, and resuspended in 6 mL complete growth medium. One quarter of the cell suspension volume was re-plated in a 6-well, incubated for 24 h, and treated with cisplatin at either the IC_50_ or a fixed-dose of 5 µM (IC_50_ of the resistant subclones NCCIT-R and 2102Ep-R) for another 48 h. (ii) In order to assess possible long-term effects of low-dose 5-aza, cells were treated in the same way except for a prolonged drug-free period of 7 days (168 h) between the end of 5-aza treatment and onset of cisplatin exposure. Cell viability was assessed by both the trypan blue staining assay and a semi-automated cell counting using a Beckman Coulter Vi-CELL (Beckman Coulter, Krefeld, Germany). Each experiment was performed in triplicates and repeated independently at least two times.

### 4.4. Flow Cytometry

For cell cycle analysis, 5 × 10^3^ cells of each cell line were treated with or without 5-aza and then analyzed by flow cytometry (FACS Calibur, BD Bioscience, Bedford, MA, USA) after propidium iodide (PI) staining as described before [36] Cells were analyzed after (i) 72 h of 5-aza treatment and (ii) a 168h drug-free period following an initial 72 h exposure to 5-aza in order to determine 5-aza-related long-term effects on cell cycle distribution (Figure 1). At least three different experiments for each treatment were conducted.

### 4.5. Western Blotting

Whole cell extracts were isolated using RIPA buffer (150 mM NaCl, 50 mM Tris-HCl pH 7.6, 1% Nonidet P-40 (*v/v*), 0,25% NaDesoxycholat (10%) (*w/v*), 1mM Phenylmethylsulfonylfluorid (PMSF), 1mM Natriumorthovanadat (Na_3_VO_4_), 10 mL Complete Protease Inhibitors (Aprotinin/Leupeptin)). Equal amounts of total protein (30μg) were electrophoresed using sodium dodecyl sulfate polyacrylamide gel electrophoresis (SDS-PAGE), all as described before [37]. Immunoblot analysis was performed with the following antibodies: caspase-3 antibody (Cat.-No. 9665S, Cell Signaling, Danvers, MA, USA) and PARP antibody (Cat.-No. 9542S, Cell signaling) for assessment of apoptosis induction. β-actin (antibody Cat.-No. sc-47778, Santa Cruz Biotechnology, Dallas, TX, USA) served as a loading control.

### 4.6. Statistical Analysis

Statistical analyses were performed using GraphPad Prism software version 5.01 (GraphPad Prism software Inc., La Jolla, CA, USA). The statistical significance of differences in cell viability and in the cell cycle phases distribution was calculated using two-sided, unpaired student’s *t*-test. A *p*-value < 0.05 was considered as statistically significant.

## 5. Conclusions

In summary, we report here that 5-aza not only effectively affects cell viability and strongly induces apoptosis at low nanomolar concentrations as a single agent, but also achieves a substantial re-sensitization towards cisplatin in an in vitro EC model of cisplatin-resistance supporting the evaluation of low-dose DNMTIs in cisplatin-resistant GCTs in the setting of a clinical study.

## Abbreviations

5-aza	5-azacitidine
5-mC	5’-methyl cytosine
CDDP	Cisplatin
DAC	5-aza-2’-deoxycitidine, decitabine
DNMT	DNA methyl transferase
DNMTI	DNA methyl transferase inhibitor
EC	Embryonal carcinoma
FDA	Food and Drug Administration
GCNIS	Germ cell neoplasia in situ
GCT	Germ cell tumor
PARP1	Poly-(ADP-ribose) polymerase 1
